# A data fusion approach to the estimation of temporary populations: An application to Australia

**DOI:** 10.1371/journal.pone.0259377

**Published:** 2021-11-11

**Authors:** Elin Charles-Edwards, Jonathan Corcoran, Julia Loginova, Radoslaw Panczak, Gentry White, Alexander Whitehead

**Affiliations:** 1 Queensland Centre for Population Research, the University of Queensland, St Lucia, QLD, Australia; 2 Centre for Data Science, Queensland University of Technology, Brisbane, QLD, Australia; ISI Foundation: Fondazione ISI - Istituto per l’lnterscambio Scientifico, ITALY

## Abstract

This study establishes a new method for estimating the monthly Average Population Present (APP) in Australian regions. Conventional population statistics, which enumerate people where they usually live, ignore the significant spatial mobility driving short term shifts in population numbers. Estimates of the temporary or ambient population of a region have several important applications including the provision of goods and services, emergency preparedness and serve as more appropriate denominators for a range of social statistics. This paper develops a flexible modelling framework to generate APP estimates from an integrated suite of conventional and novel data sources. The resultant APP estimates reveal the considerable seasonality in small area populations across Australia’s regions alongside the contribution of domestic and international visitors as well as absent residents to the observed monthly variations. The modelling framework developed in the paper is conceived in a manner such that it can be adapted and re-deployed both for use with alternative data sources as well as other situational contexts for the estimation of temporary populations.

## Introduction

Most official population statistics produced by national statistical agencies capture the usually resident or de jure population of an area. However, given that human populations are dynamic as a function of both diurnal alongside temporary mobility—moves of one night or longer in duration that do not entail a change in usual residence [1]—these official statistics miss important temporary shifts in population numbers. These temporary movements are undertaken for diverse purposes, including tourism and work; are of variable intensity, duration, seasonality and geographies; and combine to create a dynamic national population surface [2]. A recent survey of user needs in Australia indicated that there is a need for estimates of temporary populations for a range of purposes, including (but not limited to): the planning and provision of infrastructure and services, fiscal equalisation grants, understanding economic and social impacts, emergency preparedness, and to serve as denominators for epidemiological models and a range of social statistics such as crime [3]. The rapid spread of COVID-19 infections has further underscored the need for accurate and timely information regarding collective mobility.

Until recently, temporary population estimates have been limited in both number and scope due to a reliance on input data derived from the census and surveys. The proliferation of geo-located data from mobile phones and other digital technologies has increased the production of these estimates [4] with several coordinated programs of research including ENhancing ACTivity and population mapping (ENACT) based in Europe [5,6] and the Population 24/7 project in the United Kingdom [7]. The literature on temporary populations is highly fragmented, reflecting the applied nature of this field. The geography and temporality of estimates also vary widely, reflecting the purpose for which they were generated as well as the constraints of available data [4].

There are two basic approaches to estimating temporary populations [8]: the direct and indirect approaches. The direct approach utilises data drawn directly from temporary movers via census or survey. Purposive surveys of domestic and international travel can provide high-quality data but are expensive and suffer from significant sampling variability, particularly for small geographic areas and short-temporal intervals at which temporary population estimates are required.

The indirect approach employs symptomatic data such as electricity usage, sewage data or night-lights to sense changes in the population present (both residents and visitors) and their variation over time [9,10]. Symptomatic data sets such as water or electricity usage, while providing fine-grained temporal data, are often spatially discrete and provide little or no information on the composition of populations. Furthermore, these data sets are often non-stationary over space and time as other factors such as climate and seasonal effects impact usage [11]. Symptomatic data are usually collected by companies or institutions for specific geographical areas, making it difficult to consolidate them into larger national datasets. The privacy and security of individuals remain on the forefront of electricity or sewage study concerns, in particular when using and distributing high-resolution or consumer data. When working with non-identifiable and aggregate data, studies report no concerns regarding confidentiality or privacy [12].

The distinction between direct and indirect approaches has become fuzzier over time with the emergence of new data sources such as mobile phone records [13,14] and social media data [15,16]. Geo-tagged digital records can provide insights into the locations of populations and how they shift over time [17,18]. These emerging data sets have excellent spatial and temporal coverage but often suffer from selection bias and cannot be considered fully representative [19,20]. Mobile phone data, which provide an opportunity for mapping a significant proportion of a population [13] (e.g., international visitors [21]), are not available in all jurisdictions and can miss important groups such as children and the elderly.

Accessing novel data can be challenging and expensive, with many data sets owned by private companies and not readily accessible to academics and the broader research community. These data often come in the form of already processed datasets with limited or no information regarding the methodologies of data collection and processing [22]. Moreover, companies can change the ways they collect, process, and provide access to data overnight (Twitter removed the precise geotagging feature in tweets on 18 June, 2019 [23]) affecting research methods and practices.

Using and distributing such novel data are subject to important privacy concerns over personal freedom and ethics, with much research dedicated to the important topic of geoprivacy [23,24]. Therefore, methods for extracting meaningful information from location-identifiable datasets must protect user’s privacy [25]. This can be achieved by working with anonymized and aggregated data sets in the case of social media data or using phone call activity instead of individual user’s data in the case of mobile phone data [13].

Novel data sets offer the potential for the development of innovative demographic research approaches due to their higher spatial and temporal resolution [15,16,20]. After addressing technical, computational, statistical, and ethical challenges, population counts can be produced repeatedly between data releases of official statistics and at small geographical levels [20]. Another advantage of non-conventional data sources is that they can capture forms of mobility that are not represented in the official statistics [19]. Increasingly, researchers interested in the estimation of temporary populations are adopting a data fusion approach, whereby multiple data sets are used to approximate the various components of temporary populations which are subsequently combined to derive estimates [6]. However, social media data are not standardised and were not designed to monitor population dynamics; other sources such as Airbnb occupancy provide only partial estimates of temporary populations [4].

The estimation of temporary populations is further complicated by a lack of standard concepts and metrics for the enumeration of temporary populations. Commonly used concepts include the population present in a region at a point in time, the peak or maximum population in a region over a given interval (usually a year), the total number of visitors in a given interval and person time (visitor nights) in an interval [3]. These concepts are not mathematically consistent and have different attributes including the ability to be aggregated across space and over time. For example, measures of peak population cannot be aggregated across space as the timing of population peaks will vary across regions. Similarly, estimates of total visitor numbers risk double-counting when aggregated across wide areas as visitors can be counted at both origins and destinations. Here, we prefer a ‘person time’ unit as the measure of temporary populations which can be converted to a measure of Average Population Present (APP) by dividing by the number of days in the measurement interval [3]. Person time can also be aggregated across space and over time.

This paper contributes to a growing body of research in temporary population estimation (see [4] for an overview) by developing the first set of estimates for Australian regions. Australia is a country with high population mobility, with an estimated 5 per cent of the population enumerated away from home on the night of the 2016 census [26]. A diverse array of temporary movements are undertaken in Australia with distinct spatial and temporal signatures making it an ideal and challenging case study [2]. As no single data set covers these distinct populations at the temporal and spatial scale necessary to produce temporary population estimates, it was necessary to integrate both conventional and novel data sources. The flexible modelling framework enables additional data sets to be integrated should they become available.

## Materials and methods

Ethics approval has been granted by the University of Queensland in compliance with the National Statement on Ethical Conduct in Human Research (2007) https://www.nhmrc.gov.au/about-us/publications/national-statement-ethical-conduct-human-research-2007-updated-2018. The collection of data complied with the terms and conditions from Twitter, Facebook and AirDNA.

### Data

There are three major surveys capturing temporary populations in Australia: the Australian Census of Population and Housing; the Australian National Visitor Survey and the International Visitor Survey. The quinquennial Australian Census provides a snapshot of temporary populations on a single night every five years. The most recent Census in Australia took place on 9 August, 2016 and counted 23,401,892 people [27]. This is a function of the de facto enumeration strategy which captures people where they are physically located on census night. Cross-classifying the place of enumeration with data on place of usual residence provides information on the origin, destination and attributes of temporary movers. The estimates are provided at the level of Statistical Area 1 (SA1) which are geographical areas with an average population size of 400 people (n = 57,532). The SA1s aggregate to form SA2s with a population ranging between 3,000 and 25,000 people (n = 2,310). SA3s provide regional level output and generally have a population between 30,000 and 130,000 people (n = 358).

This study uses SA3s as the spatial unit of the modelling. This scale is useful within the Australian context as it captures well seasonal variation in population numbers and can be aggregated into custom regions. The survey of user preferences has indicated that the preferred geographies were Local Government Areas reflecting their political function [3]. However, our choice was also guided by considerations of data availability and processing minimising privacy issues and reporting small numbers. The SA3s are designed with the consideration of the geographic distribution of the population in Australia [28]. Most of Australia’s population is concentrated in urban centres, particularly the capital cities, located in coastal regions in the south-east, east and south-west. In the major cities, SA3s are generally smaller in size and represent clusters of suburbs around major transport and commercial hubs. In regional areas, SA3s are larger and capture areas serviced by regional cities. And in remote areas, SA3s cover very large areas that share similar identity and socio-economic characteristics.

Despite the richness of census data with respect to the pattern of moves and attributes of movers, it provides no information on the duration, frequency or seasonality of temporary movements. This is an issue as the census is scheduled (a Tuesday night in mid-Winter) to minimise the likelihood of people being enumerated away from home. Moreover, strong seasonality in temporary movement means that the pattern and attributes of movers captured at the census will not reflect the pattern at other times of the year [14].

The Australian National Visitor Survey (NVS) [29] is a continuous telephone survey that samples 120,000 Australians aged 15 and over each year. Respondents are asked about their travel over the four weeks before the survey. The NVS data provides estimates of individual trips by the place of origin (NVS Origin) and location of destination (NVS Destination) by duration, purpose and timing on a monthly and yearly basis. The International Visitor Survey (IVS) [30] complements the NVS by providing detailed spatial information on the travel of international visitors during their time in Australia. The IVS samples 40,000 departing travellers (aged 15 and over) per annum including places visited, duration and timing of trips which are disseminated on a quarterly basis.

Both the NVS and IVS provide a richness of spatial (coded to SA2s) and temporal (monthly and quarterly respectively) detail but also have several limitations. Because these surveys are based on a sample, rather than a census, the results suffer from sampling variability as they may have a large margin of error in some areas (e.g., with poor phone coverage), particularly when cross-classifying data [2]. In addition, some forms of moves (e.g., bi-residential living arising from parental separation) are not included in the sampling population.

We systematically evaluated several novel data sources for the estimation of temporary populations in Australia including Facebook Ads data; Twitter data; and Airbnb data. Several attempts were made to access mobile phone data from several Australian telecommunication companies; however, costs were prohibitive.

Facebook [31] is one of the most widely used online social networking sites worldwide and in Australia. As of January 2020, there were an estimated 16 million active monthly Australian users, equivalent to more than 60 per cent of the total population. We draw on anonymised, publicly available and aggregated data from the Facebook advertising platform [32]. Facebook Ads data have been used to monitor stocks of international migration [33,34] and small-area population mobility [35] but, not to our knowledge, nationwide temporary populations. One strength of Facebook data is that it has a wide coverage providing a unique opportunity to map temporary populations. Facebook data were scraped using the Facebook Ads Application Programming Interface (API) for the period between 19 December 2018 and 10 April 2019 (see S1 File for further detail). It provides average daily (DAU) and monthly (MAU) average estimates of users "living in", "travelling through", and "recently in", a postcode. We sampled all categories comparing the Facebook population counts with census population counts. The "recently in" ratios were consistently higher than 1, suggesting that this category captured a population element in addition to those that lived in a given postcode. There was also more volatility in the "recently in" metric, suggesting a more direct relationship with the underlying data than "living in" which may have undergone some post-processing. Therefore, we selected "recently in" category as the best way to conceptualise the population present in a region.

Twitter [36] is a real-time social microblogging service where users post short messages which are called tweets. A geotagged Tweet is a post that contains information about geolocation as exact coordinates (latitude and longitude) or place names (e.g., city, state, or country). Geolocated Twitter data represents approximately 1% of tweets [37] and has demonstrated the potential for estimating permanent and temporary internal and international movements [16,38–41]. Overall, these studies found that Twitter can act as a reliable source for studying patterns of human mobility. We extracted Twitter data using API covering the period from 22 May 2018 until 21 May 2019, representing 9,506,128 geotagged tweets of 211,082 distinct Twitter users. We excluded Twitter data (n = 192,599 tweets, or 2.0%) with missing or incomplete spatial information for the period between 28 and 29 May 2018, and between 29 January and 25 February 2019 due to interruptions in connection to the API. Further, we excluded 940,941 tweets that showed a bot-like behaviour or were geocoded to the level of country or states (see S1 File for further detail).

There are numerous limitations of research using social media data. Facebook and Twitter data are not representative of the population as a whole and people of different sex, age, income, and place of residence use mobile phones and social media to a different extent [42]. Location can be self-reported and may not reflect the real location of the user at the time of posting [15]. There can be fake accounts, duplicates and bots. Moreover, the use of social media data comes with various ethical and data protection issues. For example, users might have limited awareness of what social media data is collected, processed and shared with third parties. To better protect individual privacy, Twitter removed the service that automatically tags precise locations in June 2019. Although missing coordinates can be inferred as for example in [43], the policies around the usage of social media data can change over time posing significant challenges for consistent and reproducible research.

Airbnb is an international online firm that connects owners of rental properties with renters. Launched in California in 2008, currently Airbnb accommodation can be found around the world. In Australia, the popularity of Airbnb has rapidly grown in the last 5 years, reaching in excess of 300,000 individual listings in 2019. Airbnb has been used for the analysis of tourism activity and planning impacts yet its application for demographic research has been limited. Monthly data on the number of Airbnb accommodations, the number of reservations and reservation days at high geographical resolution can be purchased from licensed resellers at relatively low cost. Data on the number of guests are not readily available but can be estimated based on the number of bedrooms and other attributes. The Airbnb dataset used in this research was obtained from Airdna and covers the period between 1^st^ October, 2014 and 1^st^ June, 2020 (see S1 File for further detail).

In addition to regional level data from conventional and novel data sets we utilise daily estimates of the total population present in Australia to constrain our monthly population estimates. This is calculated using a population accounting framework whereby the population stock in Australia is incremented on a daily basis by natural increase and daily international arrivals and departures (both temporary and permanent) [44]. Input data for these estimates were provided to the authors by the Australian Bureau of Statistics (ABS). The resident population component of our estimates is based on the Estimated Resident Population (ERP). The ERP represents the official measure of the Australian population which is based on the concept of usual residence [45]. The estimates capture all people who live in Australia including residents who are overseas for less than 12 months and excluding overseas visitors who are in Australia for less than 12 months. Initially calculated at the Census date, it is then backdated to 30 June of the Census year. It is updated quarterly at the national and state levels. At the level of substate geographies (SA2s), the ERPs are updated annually.

Table 1 summarises the candidate data sets described above along with the strengths and limitations of each data set. Four principal dimensions of each data are recorded, including:

1. *Spatial coverage*: the geographic extent of the data as well as the smallest spatial unit for which data are available;
2. *Temporal coverage*: time period for which data are available along with the shortest interval for which data are available;
3. *Population coverage*: the extent to which data capture the target population (i.e. visitors, residents and residents absent);
4. *Concept measured*: temporary populations can be conceptualised as the population present in a region (both residents and visitors); peak population; the number of visits or visitors; or person time, commonly termed visitor nights in the tourism literature [3].

**Table 1:** Summary of candidate data sets.

Data set	Population coverage	Spatial coverage and resolution (extent and the smallest unit)	Temporal coverage and resolution (time period and the shortest interval)	Concept measured	Strengths	Limitations
Australian Census of Population and Housing	All people present in Australia on census night	• National • SA1	• Quinquennial • Night	• Population present (de facto) • Visitors	• Excellent spatial and population coverage • Transparent methodology and documentation • Long time series of historical data	• Limited temporal coverage • Updated infrequently
NVS	Australians aged 15 and over. Excludes moves between multiple residences including second homes and FIFO	• National • SA2 (but significant sampling variability)	• Continuous • Month	• Visitors • Visitor nights	• Good temporal and population coverage • Transparent methodology and documentation	• High levels of sampling variability at the SA2 level
IVS	• Short-term international travelers aged 15 years and over	• National • SA2	• Continuous • Quarter	• Visitors • Visitor nights	• Good population coverage • Transparent methodology and documentation	• Quarterly time interval
Facebook	Users of Facebook	• National • Postcodes	• Continuous • Daily	• Population present (Estimated monthly average users)	• Real-time information • Relative ease of data extraction at no-cost • Relatively high resolution but postcode only	• Data quality issues–black-box methodology • May not be representative of the population (bias towards younger people) • Technical outages during data collection
Twitter	Users of Twitter	National Geo-located and georeferenced Tweets	• Continuous • Real-time	• Population present (Estimated monthly distinct users)	• Real-time information • High resolution for historical data, reasonably high for new ones • Relative ease of data extraction at no-cost	• May not be representative of the population (bias towards younger people) • Location can be self-reported that may not reflect the real location of the user at the time of tweeting • Technical outages during data collection • Bots and non-human accounts
Airbnb	• Users of Airbnb	• National • Point estimates	• Continuous • Month	• Monthly reservation days (a factor of visitor nights)	• High geographical resolution	• Location is slightly adjusted for security reasons • Cost • No reason for stay • No real number of guests

**Fig 1:**
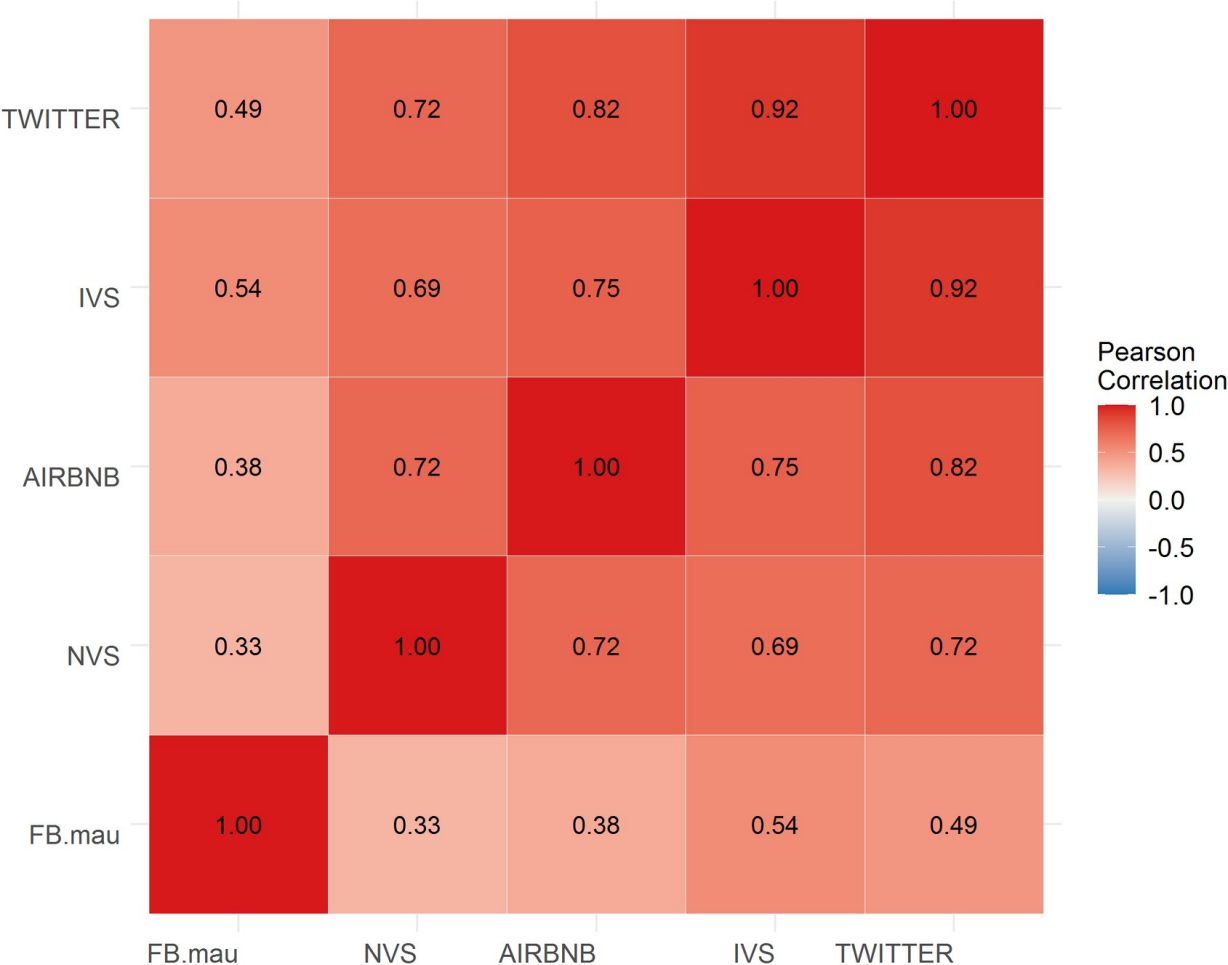
Correlation matrix heat map of candidate data sets. Source: authors’ own calculations.

A correlation heat map of the candidate data sets by month (2018) and geographic region (SA3) (Fig 1) reveals important variations in the correlation between the different data sets.

These variations reflect fundamental differences in both population coverage as well as the concepts being measured. Facebook data are the least correlated with the other candidate data sets, recording an ρ of just 0.33 with the NVS data and a maximum ρ of 0.54 with the IVS data. The poor correlation may in part reflect bias in population coverage towards younger people, although Twitter suffers from similar issues and correlates highly with all other candidate data sets. The ‘black box’ methodology used to generate Facebook estimates makes it difficult to further unpack the differences. Correlations between other data sets range from 0.92 (Twitter and IVS estimates) to 0.69 (NVS and IVS). The moderate correlation between IVS and NVS data reflects differences in the travel behaviour of domestic versus international visitors. By contrast, Airbnb data, which includes both international and domestic guests, is more strongly correlated with both IVS data (ρ = 0.75) and NVS data (ρ = 0.72). Twitter data are strongly correlated with international visitors (IVS) (ρ = 0.92) but more weakly correlated with domestic visitation (NVS) (ρ = 0.72).

Given differences in the population coverage, spatial and temporal coverage and concepts captured by the various data sets, it is necessary to adopt a data fusion approach. This data fusion approach to estimating temporary populations seeks to exploit the different strengths of the candidate data sets in a way that provides the greatest coverage (spatially, temporally and in terms of concepts captured) and thus we argue more reliable and robust set of estimates as a result.

### Modelling approach

Our model estimates the APP by month for SA3s across Australia and is outlined in Eqs 1 to 11, below. The APP is the average nightly population in a SA3 in a given month and defined as $\hat{Y}(t,s)$. It includes the resident population present, visitors (both domestic and international) and takes into account usual residents who are absent. Each of these components is estimated separately. With respect to the APP metric, it can be estimated by averaging nightly population present in a region or by dividing person nights by the number of nights in a given month. This allows us to draw upon data capturing both the population present in a region and data on person nights. Another strength of this measure is that it can be aggregated across geographic regions and averaged over longer time periods to generate quarterly or annual estimates. A limitation of the approach is that it dampens daily maxima in visitor numbers. The sum of the APP across all SA3s by month is constrained to national level estimates of the APP.

The model takes the following form. We fit a Poisson generalised linear model (GLM) for the Airbnb Visitor Nights (for 12 months of the year 2018) as a function of month, remoteness and climate. This GLM provides weights for each month-remoteness-climate combination for estimating the proportion of average overnight international and domestic visitors.

The geographic remoteness classification is based on the Accessibility and Remoteness Index of Australia (ARIA+) [46] that measures the road distance from a point to the nearest urban Centres and Localities. ARIA+ underlies the ABS’s Australian Statistical Geography Standard (ASGS) Remoteness Structure [47]: Major Cities of Australia, Inner Regional Australia, Outer Regional Australia, and Remote Australia (a combination of Remote and Very Remote Australia). This nationally consistent measure of remoteness is widely used within the Australian community. The remoteness area classification is provided for SA1s, and the SA1 population was used as the weighting unit to create the remoteness classification for each of the SA3s.

The gridded climate classification data based on temperature and humidity was obtained from the Australian Bureau of Meteorology [48]. It includes six climate zones: Hot humid summer; Warm humid summer; Hot dry summer, mild winter; Hot dry summer, cold winter; Warm summer, cool winter; and Mild warm summer, cold winter. When SA3s spanned across more than one climate zone, the classification was made based on the majority of the area covered.

The modelling approach uses the high-resolution Airbnb data to estimate weights applied to the low resolution NVS and IVS data to extrapolate monthly population estimates based on the climate and remoteness measures. Weights are calculated by fitting a GLM with a Poisson likelihood for the dependent variable, the Airbnb visitor nights counts at location *s* and time (month) *t Y*_*AirBnB*_(*t*,*s*). Variation due to month (seasonality), climate, and remoteness is accounted for by including these as independent variables in the GLM. Using an offset for the annual Airbnb visitor nights the resulting GLM yields a proportion or weight *λ*(*t*,*s*):

$$E(Y_{AirBnB}(t,s))=\lambda (t,s)\times \sum_{t,s}Y_{AirBnB}(t,s)$$

where

$$\log\left(\lambda (t,s)\right)=\beta_{0}+\beta_{1}Month(t)+\beta_{2}(Climate(s)\times Month(t))+\beta_{3}Remoteness(s)\times Month(t)-\log\left(\sum_{t,s}Y_{AirBnB}(t,s)\right)$$ (1)

The resulting weights $\hat{\lambda }(t,s)$ are estimated from the model and used to compute the monthly average over-night domestic visitors as the product of the proportion estimated from the Airbnb data ${\hat{\lambda }}_{AirBnB}(t,s)$ and the annual number of visitors according the NVS *Y*_*NVS*_ divided by the number of days per month *n*(*t*)

$${\hat{Y}}_{NVS}(t,s)=\hat{\lambda }(t,s)\times \frac{Y_{NVS}}{n(t)}.$$ (2)

Similarly, the monthly average overnight international visitors are estimated as the product of the proportion estimated from the Airbnb data ${\hat{\lambda }}_{AirBnB}(t,s)$ and the annual number of visitors according the IVS *Y*_*IVS*_ divided by the number of days per month *n*(*t*)

$${\hat{Y}}_{IVS}(t,s)=\hat{\lambda }(t,s)\times \frac{Y_{IVS}}{n(t)}.$$ (3)

A second Poisson GLM for the dependent variable is computed for the NVS Origin nights using the independent variables for the month and remoteness. The results of this model provide weights for computing the monthly average number of overnight residents domestically and internationally.


$$E\left(Y_{NVS\;Origin}(t,s)\right)=\eta_{NVS\;Origin}(t,s)\times \sum_{t,s}Y_{NVS\;Origin}(t,s)\log\left(\eta_{NVS\;Origin}(t,s)\right)=\alpha_{0}+\alpha_{1}Month(t)+\alpha_{2}Remoteness(s)\times Month(t)-\log\left(\sum_{t,s}Y_{NVS\;Origin}(t,s)\right)$$ (4)

These sample proportions ${\hat{\eta }}_{NVS\;Origin}(t,s)$ are used to calculate estimates of monthly averages on a finer-grained scale (with respect to geographical area) for the resident populations based on the IVS, NVS data. The estimated monthly average number of residents travelling domestically overnight (the usual residents who are absent domestically) is the product of the estimated weights and the total annual number of domestic visitors divided by the number of days per month

$${\hat{Y}}_{Resident\;Domestic}(t,s)={\hat{\eta }}_{NVS\;Origin}(t,s)\times \frac{Y_{NVS\;Domestic\;Annual}}{n(t)}.$$ (5)

The estimated monthly average number of residents travelling internationally overnight (the usual residents who are absent internationally) is the product of the estimated weights and the total annual number visitors divided by the number of days per month

$${\hat{Y}}_{Resident\;International}(t,s)={\hat{\eta }}_{NVS\;Origin}(t,s)\times \frac{Y_{NVS\;International\;Annual}}{n(t)}.$$ (6)

Summing these components and the ERP (monthly at the SA3 level) produces the unscaled estimate of the monthly populations for each SA3.


$${\hat{Y}}_{Total\;Unscaled}(t,s)={Estimated\;Resident\;Population+\hat{Y}}_{NVS}(t,s)+{\hat{Y}}_{IVS}(t,s)-{\hat{Y}}_{Resident\;Domestic}(t,s)-{\hat{Y}}_{Resident\;International}(t,s).$$ (7)

Next, we compute the monthly differences between the national monthly populations means (provided to the authors by the ABS) and the sum of the unscaled present (monthly) populations.

The difference between the overall population monthly means and the unscaled estimates is used to produce monthly differences between all SA3s and national monthly estimates.


$$\Delta_{Total}(t)=AverageMonthlyPopulation-\sum_{s}{\hat{Y}}_{Total\;Unscaled}(t,s)$$ (8)

This difference is multiplied for each SA3 by the proportion of the unscaled SA3 populations for each SA3.


$$SA3_{proportion}(s)=\frac{{\hat{Y}}_{Total\;Unscaled}(t,s)}{\sum_{s}{\hat{Y}}_{Total\;Unscaled}(t,s)}$$ (9)

Finally, this population adjustment is added to the unscaled population estimate for each SA3 and month resulting in an estimate of the average population present (APP) for each SA3 and month.


$${\hat{Y}}_{Total\;Scaled}(t,s)={\hat{Y}}_{Total\;Unscaled}(t,s)+PopulationAdjustment(t,s).$$ (10)

These resulting estimates are scaled estimates of the monthly population of each SA3 taking into account all seasonal variation.

All analyses and visualisations were performed using R version 4.0.2 by R Core Team [49] using the tidyverse [50], tmap [51] and lme4 [52] packages. Data and R scripts are available for download using the following link: https://osf.io/f2nhs/?view_only=af8e843bc2e546408ead543d72da23e5.

## Results

**Fig 2:**
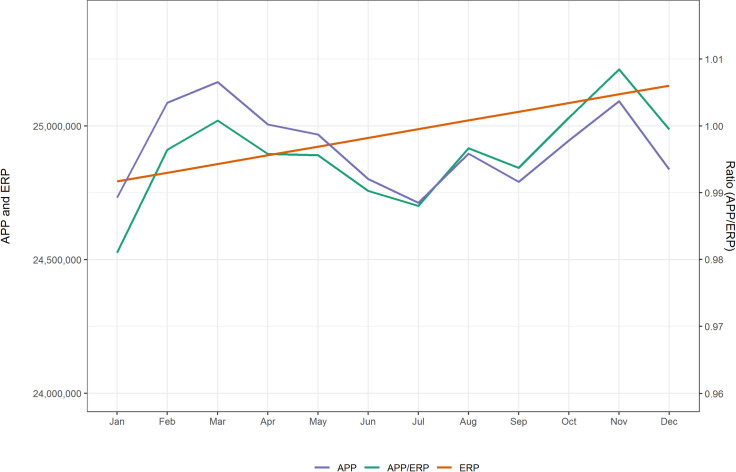
Population physically present and ERP, Australia, 2018. Source: authors’ own calculations.

Fig 2 shows the national level estimates of the APP and the ERP for 2018. The population physically present in Australia is higher than the ERP in February, March and April but lower than the ERP in all other months due to more residents being absent overseas than overseas visitors present. The nadir is in January and July 2018 when the APP is 1 per cent lower than the ERP. This corresponds to Australian school holiday periods. These national level estimates of the population physically present are used to constrain the SA3 level estimates.

**Fig 3:**
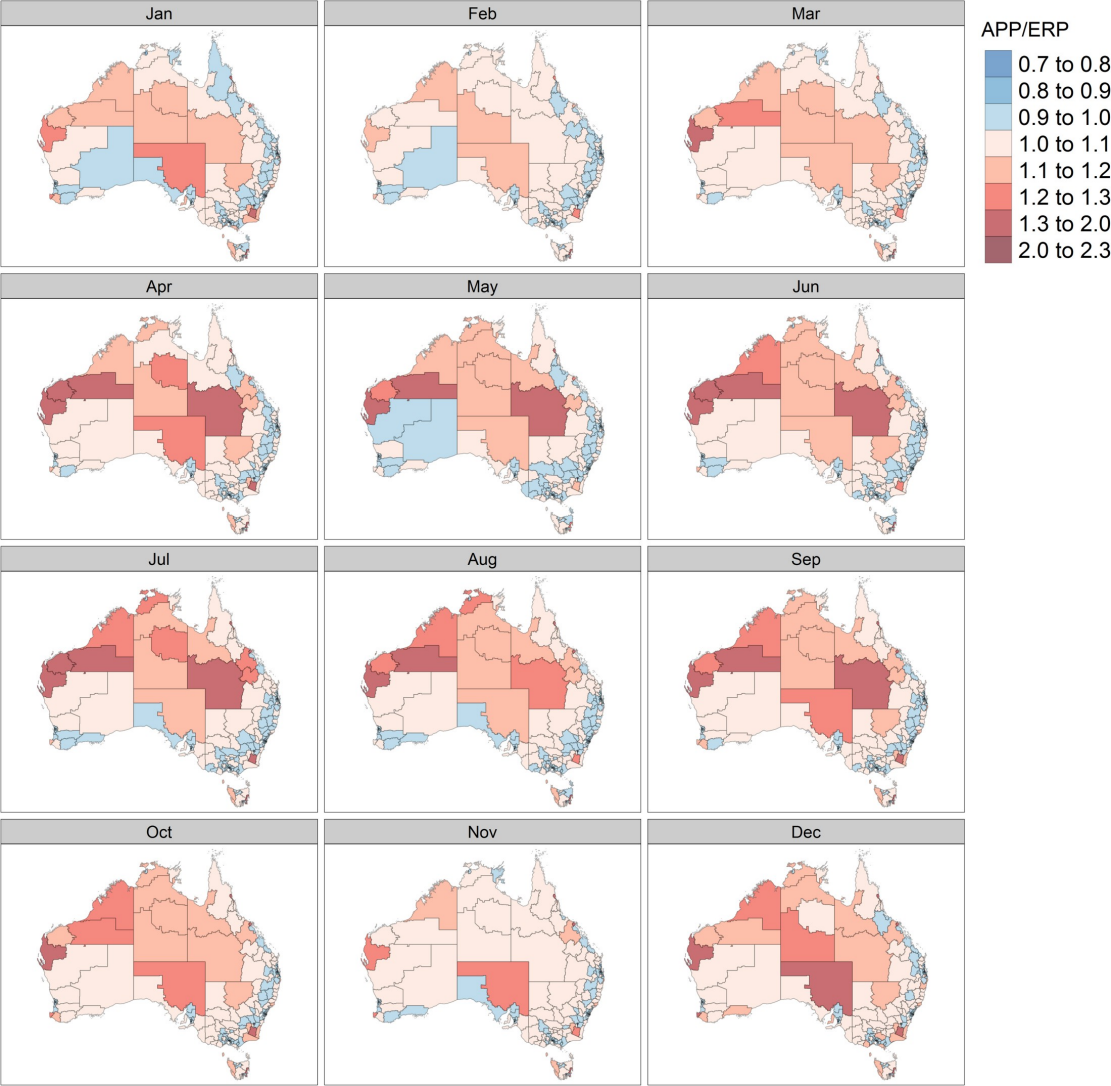
Monthly variations in ratio of the APP to ERP across SA3 regions in Australia. Source: authors’ own calculations. The maps were created using tmap package in R [51]. Freely available digital boundaries for SA3s were obtained from the ABS: https://www.abs.gov.au/websitedbs/d3310114.nsf/home/digital+boundaries.

We calculated an APP for each SA3 in Australia by month (only one SA3 the Illawarra Catchment Reserve was excluded from the analysis due to low numbers of residents and inconsistences between the ERP and results of visitor surveys). The ratio of the APP to the ERP reveals important differences between our ‘dynamic’ APP estimates and the ‘static’ ERP. The results are estimated monthly in order to observe fluctuations in resident populations across SA3s throughout 2018 (Fig 3) where shades of red indicate where the APP is higher than the ERP and the opposite case is represented by shades of blue. It is important to note that the use of APP as a metric dampens short term maxima and minima, for example weekend peaks.

Several SA3s show consistently high APP/ERP ratios over the course of the year. These includes the centre of capital cities, remote mining regions and selected tourist locations. Other regions consistently record low APP/ERP ratios, i.e. the APP is lower than the ERP—these include suburban areas of the major capital cities as well as areas located in the rural hinterland of major capitals. The APP demonstrates clear seasonality in several locations. Among 335 SA3s, 253 (75 per cent) SA3s are characterised by a summer peak, and 82 (24 per cent) exhibit a winter peak. The former is concentrated in southern and coastal parts of Australia, while the latter include alpine ski resorts as well as SA3s located in central and northern Australia. In the major cities, population fluctuation reflects the timing of school holiday and public holiday periods. The APP dynamics of all Australian SA3s can be examined on the interactive dashboard available using the following link: https://qcpr.github.io/tempo/. The estimates can be downloaded and aggregated to custom geographies.

**Fig 4:**
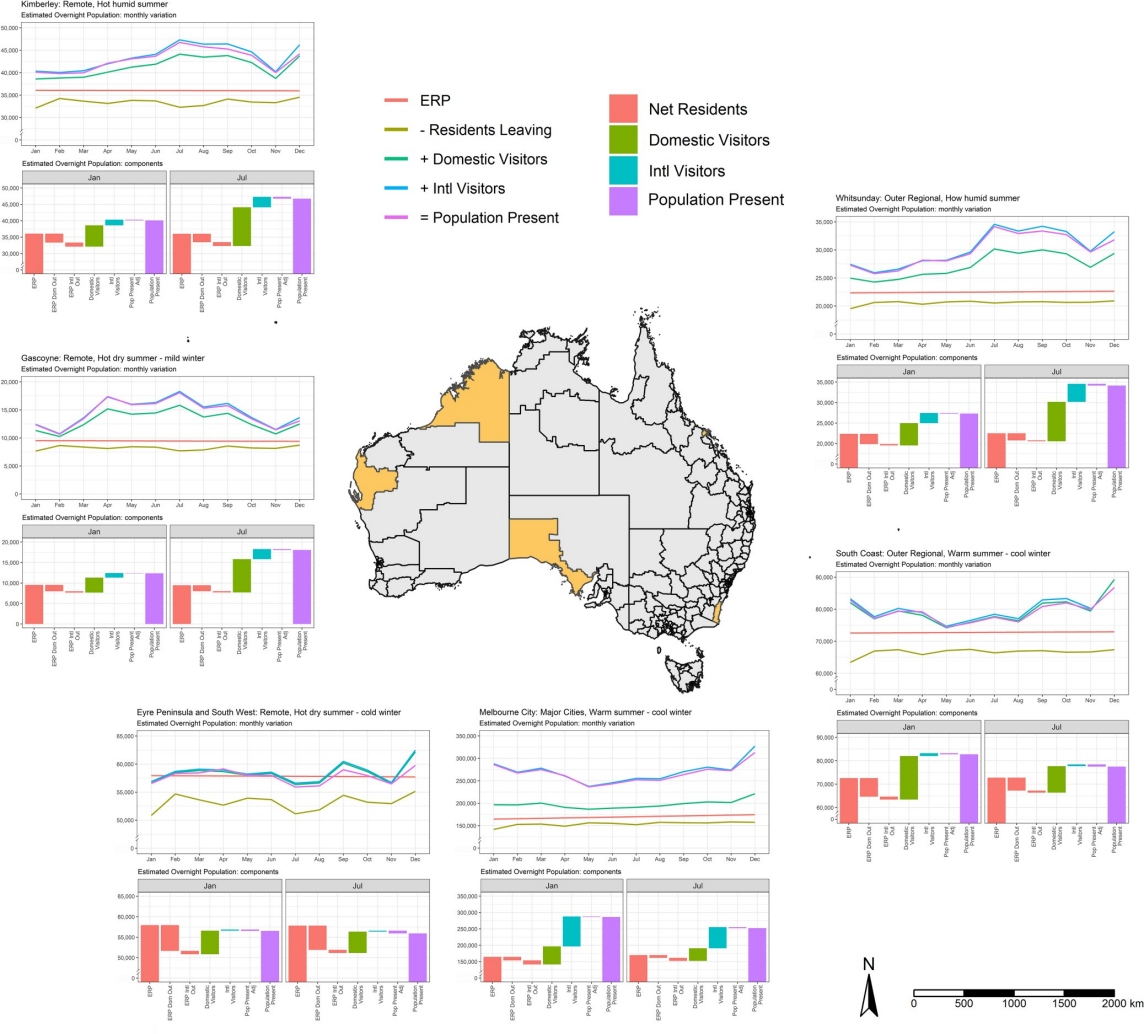
Average population present (APP), selected SA3s, Australia, 2018. The maps were created using tmap package in R [51]. Freely available digital boundaries for SA3s were obtained from the ABS: https://www.abs.gov.au/websitedbs/d3310114.nsf/home/digital+boundaries.

Fig 4 shows the seasonal variation of APP estimates for a sample of SA3s over the course of the year along with the contribution of the different components driving these shifts. Absent residents reduce the APP in all SA3s during the course of the year, with the impact most marked in January, coincident with the summer holiday period, as usual residents travel elsewhere in Australia and overseas. Both the timing and relative contribution of domestic and overseas visitors to the APP varies across the country. SA3s in northern Australia including the Kimberley (1); Gascoyne (2) and Whitsundays (6) are characterised by a winter peaks in visitation. Domestic visitors make the largest contribution to the APP, although there is a significant international component. SA3s in southern Australia (Eyre Peninsula (3); Melbourne City (4); South Coast (5)) are characterised by summer peaks along with smaller peaks corresponding to the school holiday periods ((4) and (5)). International Visitors are a major contributor to the APP in Melbourne City (4) but are less important in the Eyre Peninsula (3) and South Coast (5).

## Discussion

The aim of the current study was to establish the first set of temporary population estimates for Australia drawing on conventional (census and visitor surveys) and novel (short-term accommodation data and social media data) data sets. These novel estimates provide new insight into how population distribution in Australia changes through time. They demonstrate that even with uncertain data, temporary seasonal patterns can be observed in population movement. Our estimates captured well both the timing (winter or summer peaks) and relative contribution of domestic and overseas visitors as well as absent residents. These estimates can inform future research and policy needs where information on temporary changes in population is useful, including disaster and public health management, the planning of infrastructure, provision of goods and services, and analysis of various social and economic impacts [3]. They can also serve as denominators for a range of social and epidemiological statistics [3]. These estimates are the first of their kind in Australia and complement ongoing programs of work produced in several countries [4].

This study used a combination of official statistics, results of national and international visitor surveys and short-term accommodation data. This hybrid data fusion approach sought to exploit the strengths of the various data sets. The survey data provides accurate, unbiased data at the relatively low spatial and temporal resolution, while the short-term accommodation data (in our case Airbnb data) captured temporal trends and spatial patterns at the high resolution needed for our APP estimates. The approach enabled different components of the APP to be estimated including absent residents, domestic and international visitors.

The study also explored the potential of social media data. Previous research reported that Twitter and Facebook data offer near real-time spatially explicit geo-data in addition to being accessible and cost-efficient [18,39,41]. However, the decision was made not to use them in light of several limitations including sampling and location biases, risks of technical outages during data collection, and dependence on data reliant on what is accessible through the API. Moreover, Facebook was poorly correlated with all other data sets and there was a lack of insight into exactly what was being captured due to the “black box” method employed. Twitter data performed better but was not included in the model due to the behavioural component inherent in the data, such as the tendency for media users to post less when traveling and on holidays. However, it is unclear if results would be different elsewhere, since social media usage is known to vary across countries [18,53]. Using mobile phone data in our case was not a financially viable option. An additional difficulty is designing a reliable and reproducible data collection and preparation strategy, as the platforms are dynamic systems (data reporting and formats can quickly change).

Following a systematic evaluation of candidate data sets, we fitted a GLM for the Airbnb Visitor Nights (person time) as a function of month, remoteness and climate, providing estimates of average overnight visitors for each combination of month, remoteness and climate. In this way, the high temporal resolution of the Airbnb data is applied to the lower resolution NVS and IVS data enabling extrapolation of monthly population estimates. NVS and IVS data were used to estimate the absent resident population. The resultant estimates of the monthly APP were constrained to national estimates of the physically present population to create a consistent set of estimates for all regions across the country.

The limitations of the framework include relatively large aggregate spatial units (SA3s) and lack of measures of uncertainty. Moving beyond the current modelling framework and subject to the availability of alternative data (such as mobile phone records) new opportunities to explore finer spatial output units would be possible. The lack of uncertainty measures may act to limit the inferential possibilities of the model, but importantly do not impact the accuracy of the estimates or parameters. In terms of introducing measures of uncertainty, exploring the use of a Bayesian hierarchical model, that would return uncertainty measurements for all the parameters (and predictions) would be an interesting avenue for future work. That said, one drawback from this modelling strategy is that they are computationally intense and may not be tractable in this instance, or practical in terms of the time to implement the results. Furthermore, the current modelling framework is well within the reach of other researchers and practitioners without the need for the specialised knowledge necessary to implement a full Bayesian hierarchical model.

Future work should continue to examine the potential of geo-social and phone record data for improving temporary population mapping [14,21,54]. For example, Tweets can be integrated as a covariate layer into a census data gridded disaggregation model [18]. Even though Twitter has removed precise geotagging in tweets, research methods can be responsibly adjusted to continue research using twitter data, for example leveraging geoparsing [23]. There is an opportunity for the integration with other spatial datasets indicative of human activity, such as wifi network usage [55] and settlement, land use and built-area datasets [56]. While such integration can rely on census data (top-down), census-independent (bottom-up) approaches can be employed in scarce-data environments [57].

The estimation of temporary populations is a topic of growing international interest, especially in the context of public health, disaster management as well as scarce-data environments. A key limitation, however, is the inability to validate estimates due to the absence of a ‘gold standard’ data set capturing temporary populations. The widespread dissemination of estimates is an important first step in validating and improving data and methods for the estimation of temporary populations.

## Supporting information

S1 File Methodology and data. (DOCX)
