# Supplementary material for: A data fusion approach to the estimation of temporary populations: An application to Australia
Source: PLoS One. 2021 Nov 11;16(11):e0259377. doi: 10.1371/journal.pone.0259377 (PMC8584718; doi:10.1371/journal.pone.0259377)
Supplement: S1 File — (DOCX) [file pone.0259377.s001.docx]

**Supporting information: Methodology and data.**

Here, we describe our methodology for data collection, exploration and preparation. This includes description of the data sets and strategies for data scraping and cleaning. In this way, we follow standards for the reporting of social media and digital research [1].

**The Twitter data set**

***Data collection***

The Twitter data was scraped using Twitter’s Streaming Application Programming Interface (API) [2] which is a publicly accessible interface for third-party software programs allowing to collect a real-time stream of tweets from the Twitter platform. The Python script scraping the data ran from 22 May 2018 until 21 May 2019 to fetch data at regular time intervals. The algorithm filtered data to include geotagged tweets that were reported to occur within a specific geographical area of Australia (box bounded by 113.15, -43.6411, 153.633, -10.68).

***Data exploration***

The scraped Twitter data contains information on individual tweets, including the User ID, the status ID, location information, and the time stamp. We scrapped 9,506,128 geotagged tweets from 211,082 unique Twitter users that span the period from 22 May 2018 to 22 May 2019. Twitter data is missing/incomplete for the period between 28 and 29 May 2018, and between 29 January and 25 February 2019 (therefore, February was excluded from the analysis). Omissions have occurred due to interruptions in connection to the API and other errors. Tweets with incomplete and missing spatial data were excluded from the analysis (n=192,599 tweets). A small number of users demonstrated a bot-like behaviour (very frequent or regularly updates), leading to 132,399 tweets of 110 bot-like users being excluded. Additionally, 32,947 ‘high speed’ tweets of 287 users were excluded as the time difference and distance between consecutive tweets was unrealistic (more than 10,000 metre per second). Further, we excluded tweets originated from other countries (n=32,887 tweets in 32,703 distinct locations not overlaying shape of Australia with 0.5-degree buffer around it). Only 876,143 tweets in 139,045 distinct locations within Australia had precise geographic coordinates. A large number of tweets (n = 7,676,032, or 80.7%) had no coordinates but were labelled with ‘name of place’ with varied quality and geographical precision: 95.66% of these tweets represent the city level; 2.95% the neighbourhood level, 1.38% the level of points of interest, 0.01% the level of administrative region, and 289 tweets the level of the country. These places were geocoded using OpenCage R package [3]. Locations with the type of place corresponding to ‘country’ or ‘admin’ (states and territories) were excluded to increase the accuracy of the data. Time zones of tweets were updated to UTC time. After the cleaning, 8,372,588 tweets (88.1% of the raw data set) were included in the data set.

***Data preparation***

The geocoded point Twitter data was aggregated at the level of SA3s and SA2s. A variable “Average daily users” was calculated as the mean number of distinct daily users per month. “Monthly distinct users” was calculated as the number of distinct users who tweeted in a particular month.

**The Facebook data set**

***Data collection***

The Facebook data was collected using the Facebook ‘s advertising platform, Adverts Manager [4], that allows access to information about users to whom an ad was shown. The algorithm requires login credentials to Facebook, a business account, and a registered mobile phone number. Testing and development occurred between 3 August and 18 December 2018, with sporadic and incomplete data for this period. The script was in full operation between 19 December 2018 and 10 April 2019. We collected a total of 2,468,929 records of postcode estimates in Australia.

***Data exploration***

Facebook reports a potential reach of selected audience, producing estimates of Monthly Average Users (MAU) and Daily Average Users (DAU) at the postcode level. Locations are assigned to users based on their information and activity on their public profile, as well as device and connection information. The MAU and DAU estimates were collected for each postcode in Australia (based on the 2016 Australian Statistical Geography Standard (ASGS) non-ABS structures [5]) at regular intervals under the location classifications of 1) users who were “recently” in the area; 2) users who were “traveling” through the area; 3) users who “live” in the area; and 4) “everyone” who is in the area.

***Data preparation***

The postcode-level data were aggregated to the level of SA2 and SA3 using ASGS correspondences. Postcodes 9494 (No usual address), 9797 (Migratory - Offshore - Shipping), 2899 (Norfolk Island), 6798 (Christmas island), and 6799 (Cocos Islands) were excluded from the data set due to lack of data.

**The Airbnb data set**

***Data collection***

Data pertaining to Airbnb [6] were purchased from a third-party service for short-term rental analytics, AirDNA [7]. Airdna is a firm that generates and provides access to data on Airbnb’s performance. Its algorithm discerns whether specific Airbnb rental properties are available, reserved for occupancy, or blocked by the owner and unavailable for rental. The data contains records of 543,883 listings in Australia, covering the period from 01.10.2014 to 01.06.2020.

***Data exploration***

In the Airbnb data set, each record has 32 attributes. The attributes considered valuable for our analysis were property ID, property type, bedrooms, listing type, reporting month, occupancy rate, number of reservations, reservation days, available days, blocked days, country, state, city, zipcode, neighborhood, latitude, longitude, and whether the listing is active or not. A listing is considered to be active if it has been posted in the last 60 days or had a reservation in the last 30 days.

***Data preparation***

The geographical point data of each Airbnb listing was aggregated to the levels of SA2s and SA3s. Number of guest nights occupied was calculated using number of bedrooms (one bedroom equals one person) and reservation days.

**References:**

1. Driscoll K, Walker S. Big data, big questions| working within a black box: Transparency in the collection and production of big twitter data. J Commun. 2014;8:20.

2. Twitter 2018. Available from: <https://developer.twitter.com/en/docs>.

3. Salmon M. Opencage: Interface to the OpenCage API (R package version 0.1.4.) 2018. Available from: <https://CRAN.R-project.org/package=opencage>.

4. Facebook. Advertising on Facebook | Facebook Business 2019 [1 December 2018]. Available from: <https://www.facebook.com/business/products/ads>.

5. ABS. 1270.0.55.001 - Australian Statistical Geography Standard (ASGS): Australian Bureau of Statistics; 2016. Available from: <https://www.abs.gov.au/websitedbs/d3310114.nsf/home/australian+statistical+geography+standard+(asgs>).

6. Airbnb, Inc. 2021. Available from: <https://www.airbnb.com.au/>.

7. AirDNA: Short-term rental analytics 2021. Available from: <https://www.airdna.co/>.
